# Effect of Accumulation of Heavy Metals in the Red Fox Intestine on the Prevalence of Its Intestinal Parasites

**DOI:** 10.3390/ani10020343

**Published:** 2020-02-21

**Authors:** Marie Borkovcova, Vladimir Fiser, Martina Bednarova, Zdenek Havlicek, Anna Adámková, Jiri Mlcek, Tunde Jurikova, Stefan Balla, Martin Adámek

**Affiliations:** 1 Department of Food Analysis and Chemistry, Tomas Bata University in Zlin, 760 01 Zlin, Czech Republic; entomophagy@seznam.cz (M.B.); aadamkova@utb.cz (A.A.); 2 Infrastructure Department, Mendel University in Brno, 61300 Brno, Czech Republic; vladimirfiser51@gmail.com (V.F.); martina.bednarova@mendelu.cz (M.B.); 3 Department of Morphology, Physiology and Animal Genetics, Mendel University in Brno, 61300 Brno, Czech Republic; zdenek.havlicek@mendelu.cz; 4 Institute for Teacher Training, Constantine the Philosopher University in Nitra, 949 74 Nitra, Slovakia; tjurikova@ukf.sk (T.J.); sballa@ukf.sk (S.B.); 5 Department of Microelectronics, Brno University of Technology, 616 00 Brno, Czech Republic; adamek@feec.vutbr.cz

**Keywords:** accumulation, metals, red fox, intestinal parasites

## Abstract

**Simple Summary:**

Heavy metal pollution of environmental ecosystems has become rather a significant factor in assessing them, as heavy metals can significantly influence animal health. The objective of this study was to examine a possible association between contents of selected heavy metals such as cadmium, copper, lead, chrome, zinc, and manganese in intestines of foxes and between prevalence of fox intestinal parasites. The association was not fully proven. On the contrary, sensitivity of parasites to cadmium was demonstrated; with increasing cadmium content in the intestine of the host, prevalence of parasites decreased to zero. No parasites were found in the intestine, when concentration of accumulated cadmium exceeded the level of 0.05 milligrams per kilogram, which represents the limit for meat (excluding offal) of bovine animals, sheep, pig, and poultry according to the Regulation (EU) No. 488/2014 amending the Regulation (EC) No. 1881/2006). Thus, even cadmium content below the above limit showed an impact on parasite biodiversity.

**Abstract:**

The aim of this study was (i) to compare levels of accumulated heavy metals in the fox intestines with and without parasites. Moreover, our research also dealt with (ii) examination of the relationship between heavy metal content in fox intestines and between the presence of fox intestinal parasites. The intestines of 34 hunter-killed foxes were dissected to detect the occurrence of parasites. In 15 intestinal samples, parasitic intestinal helminths were found. Heavy metal content in small intestine tissue and in parasites was determined using atomic absorption spectrometry (AAS). The prevalence of parasites was significantly dependent on Cd content in the host’s small intestine (*p* < 0.01). To conclude, the authors suggest that parasites are sensitive to Cd levels; their prevalence in the intestines of the fox host decreases to zero with increasing Cd content.

## 1. Introduction

Wildlife, farm animals, and humans are exposed to increasing environmental pollution. The sooner this pollution is detected, the sooner measures necessary to prevent and eliminate it can be taken. Therefore, scientists have continuously been searching for new species that could be used as pollution markers [1]. Accumulation indicators are organisms, which have sufficient size, are easily available, and widely spread. They are also expected to show high accumulation potential. Moreover, pollutant contents in their bodies and average pollutant concentrations in the environment are required to correlate identically at all locations and conditions [1]. Due to their functional influence in animal hierarchy [2], that is because of their position in the food chains [3], parasites exert the potential to be regarded as bio-indicators of heavy metals, especially when anthropogenic pollution must be monitored [1,4]. Due to their higher potential to accumulate heavy metals before contamination occurs in animal tissues used for human consumption, parasites are considered bio-indicators capable of indicating possible environmental burdens. Several studies have already reported research on the use of parasites as indicators of environmental quality [5]. Specific animal indicators for detection of pollution in aquatic ecosystems have already been proposed; amounts of heavy metals found in the *Hysterothylacium aduncum* nematode were used to assess the contents of waterborne contaminants [6]. Moreover, the rat-parasite scheme [7,8] has been proposed for monitoring pollution in urban ecosystems. For other ecosystems, appropriate indicators are expected to be found.

However, the amount of heavy metals that can be tolerated by parasites has not been determined yet. Many studies [1,8,9,10] have demonstrated higher levels of heavy metals found in parasites rather than in their hosts; some researchers report objective findings on bio-remediation ability of parasites to relieve their hosts of the heavy metal burden [11,12,13].

The above findings might lead to the following presumption: by deliberately infecting a host with a parasite, the host could recover from intoxication. Another hypothesis suggests alternatively that higher concentrations of heavy metals in the tissues of a host may exert a negative effect on its parasites. Many heavy metals are toxic; a study on the effect of cadmium on the reproductive capacity of *Caenorhabditis elegans* proved damage of its intestinal lining followed by eating disorders and reduced fecundity after exposure to higher doses of Cd [14]. Regarding influence of environmental pollution, changes in populations of parasites and in their communities have often been analysed. An article summarizing findings of many studies shows that environmental pollution affects parasite populations and their communities directly or through intermediate hosts and host effects [1].

The aim of this study was (i) to compare levels of accumulated heavy metals in the fox intestines with and without parasites. Moreover, the research also dealt with (ii) examination of the relationship between heavy metal contents in fox intestines and presence of intestinal parasites.

## 2. Materials and Methods

### 2.1. Foxes

In the period of 2015 to 2018, samples of intestines of 34 red wild foxes were collected occasionally during hunts in various parts of the Czech Republic. No animal investigated in our study was kept in experimental breeding colonies. All the fox specimens were shot down as nuisance wildlife management in compliance with the Czech Act No. 449/2001 Coll. in reading of further prescriptions. Intestines of hunter-killed animals were subjected to the research.

The foxes were eviscerated in situ and the viscera were transported to a laboratory. In the laboratory, the samples were being kept at −78 °C for one month to inactivate any potential infections. After thawing, the viscera were divided into individual parts during visceral dissection. For the purpose of this study, the small intestine was used. It was divided into duodenum, jejunum, and ileum. Each part was washed separately with distilled water and stored in plastic vials containing 80% alcohol. Endoparasites were examined microscopically considering also the washed small intestine content. Although Protozoa representatives were also detected, only the species that could be further investigated by the chosen method were examined within this study. The parasites were also washed with distilled water and separately stored in plastic vials with 80% alcohol.

In 15 intestinal samples, parasitic intestinal helminths of the phylum Nematoda (roundworms) and Cestoda (tapeworms) were found. Nine samples contained tapeworms of the genus *Mesocestoides.* Helminths were not detected in 19 tissue samples of fox intestines.

### 2.2. Samples

For further processing, the biological material was categorized into four main groups:

- Small intestine (jejunum) without ingesta from foxes where no parasites were detected;
- Small intestine (jejunum) without ingesta from foxes where parasites were detected;
- Parasites, further classified into the following three groups: (a) Nematoda, (b) Cestoda—*Mesocestoides* spp., (c) Cestoda—others;
- Standard reference material (CRM 12-02-01 Bovine Liver) from the Czechoslovak Institute of Metrology.

### 2.3. Sample Analysis

For determination of the Pb, Cd, Ni, and Cr elements, atomic absorption spectrometry (AAS) with electro thermal atomization (ETA AAS) was used (detection limit: Pb 0.100 μg kg^−1^, Cd 0.005 μg kg^−1^, Cr 0.020 μg kg^−1^, Ni 0.080 μg kg^−1^). For Cu, Mn, and Zn analysis, flame atomization (FAAS) with C_2_H_2_/air flame was employed. An AAS spectrometer AA 30 with a GTA 96 graphite cuvette (Varian Ltd., Mulgrave, Australia) was used for measurement (detection limits: Cu 2.0 μg kg^−1^, Zn 10.0 μg kg^−1^, Mn 1.8 μg kg^−1^). Standard solutions of Cd, Pb, Cr, Cu, Zn, Mn, and Ni such as TraceCERT^®^ (Sigma Aldrich, Prague, CZ) and ASTASOL (Analytika, Prague, CZ) were used in construction of calibration curves for the measurements.

For background correction, Pb and Cd signals were corrected with a D2 lamp and a matrix modifier (Mg(NO_3_)_2_ + NH_4_H_2_PO_4_). Ascorbic acid was used as a matrix modifier for determination of Cr.

AAS spectrometry was employed to analyze mineralized samples prepared by wet digestion in an ETHOS SF microwave mineralizer (Milestone, Sorisole, Italy).

For mineralization, concentrated HNO_3_ was distilled using a BERGHOF equipment. Then, 0.16–0.6 g of tissue with addition of 5 mL HNO_3_ + 5 mL H_2_O (DEMI) was mineralized consequently.

The temperature program was set to reach 210 °C at maximum. After cooling, the samples were filtered into 25 mL volumetric flasks. Element concentrations were determined in µg L^−1^ and recalculated to mg kg^−1^ in wet weight. The accuracy of the analytical method was enhanced by simultaneous analysis of certified CRM 12-02-01 reference material (Bovine Liver). Levels of all determined elements ranged within the confidence interval given by the CRM producer for these elements.

### 2.4. Statistical Analysis

The obtained results were processed by the Statistica ver. 10 statistical program from StatSoft (StatSoft Czech Rep. s.r.o., Zličín, CZ) and analysis of one-dimensional data was used. Exploratory data analysis indicated an asymmetrical distribution of the acquired data. Therefore, incorrect values of a classical and robust estimation of parameters were expected. Consequently, the Box-Cox transformation method was used. Conversion of the data resulted in improved parameters of distribution. Thus, the re-expressed estimate x̅_R_ of Box-Cox transformation is one the most reliable point estimates of the centre.

In case of small samples (4 < *n* < 20), use of the Horn procedure of pivot measures is recommended [15]. Both pivot half-sum P_L_ and pivot range R_L_ are robust against outliers in a small sample. Interval estimate suggests a range of possible values with a pre-chosen probability (lower limit L_L_ < µ < upper limit L_U_). For significance level α = 0.05, the 95% confidence interval of the measure of location µ was calculated.

P_L_ − R_L_. t_L_, _0,975_ (n) < µ < P_L_+ R_L_. t_L_, _0,975_ (n) (1)

where t_L_, _0,975_ (n) = Horn’s quantile.

To evaluate the significance of variability sources in the data, non-parametric analysis of variance, namely the Kruskal-Wallis test was used.

The comparison of two independent variables such as the content of heavy metals in fox intestines containing parasites, and the content of heavy metals in fox intestines without parasites was done using the non-parametric Mann-Whitney U test.

## 3. Results

The effect of intestinal fox parasites on accumulation of heavy metals in the small intestine of foxes was evaluated by comparing detected contents of heavy metals in fox intestines with parasites with those found in fox intestines free of parasites (Table 1). All results are calculated on a wet matter basis.

Concentrations of Pb, Cr, Cu, Zn, Mn, and Ni in the parasites free intestines did not differ significantly from levels of the above heavy metals in the intestines with detected parasites (*p* > 0.05). However, concentration of Cd in the intestines of foxes without parasites (0.029 mg kg^−1^) was higher than that found in the intestines of animals with parasites (0.009 mg kg^−1^), the difference was statistically significant (*p* = 0.014).

**Figure 1:**
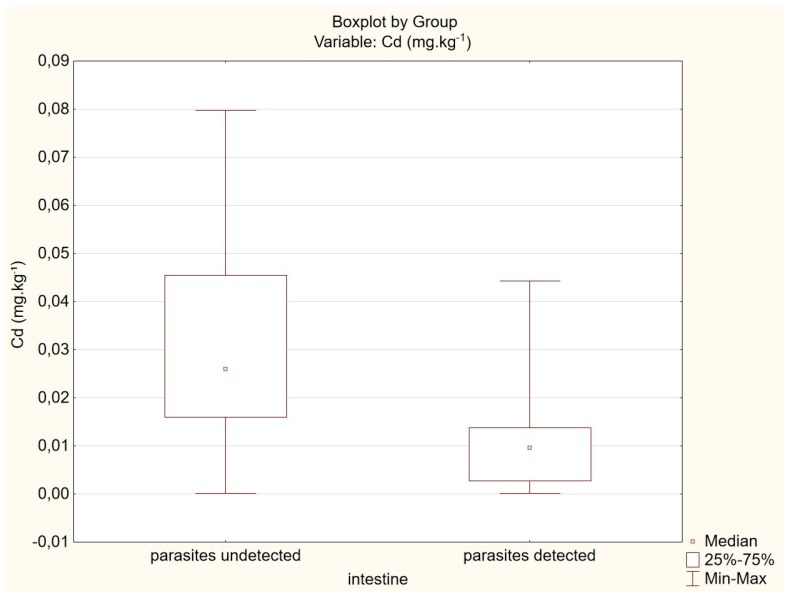
Association between prevalence of parasites and Cd content in fox intestines.

The association between prevalence of parasites and Cd content in fox intestines is shown in Figure 1.

The difference between the contents of heavy metals in parasites and between their levels in intestines of their hosts was statistically evaluated by the Kruskal-Wallis test.

**Figure 2:**
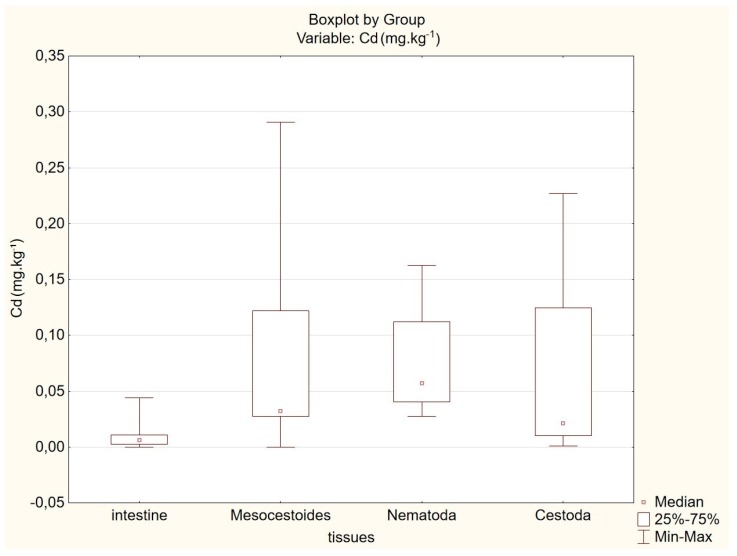
Comparison of Cd concentrations in parasite tissues with Cd concentrations in intestines of the host.

The concentrations of Pb, Cr, Cu, Zn, Mn, and Ni in parasites were higher than the concentrations of the above metals in the host intestines, and the difference between the above concentrations was statistically significant (*p* < 0.01). An exception to the above pattern is represented by Cd, for which the determined level of statistical significance ranges between the following limits: 0.05 > *p* = 0.023 > 0.01. Statistical significance lies in the uncertainty interval. Comparison of Cd contents in parasite tissues with Cd contents in intestines of the host is shown in Figure 2.

Concentrations of heavy metals in *Mesocestoides* spp. and their correlation with heavy metal contents in the intestine of the host were examined by the Spearman’s rank correlation analysis. The significance level (*p*) of the correlation was calculated. The results are shown in Table 2.

For concentrations of heavy metals in other parasite species, this correlation was not calculated due to the low number of samples (n = 4). The high Spearman’s rank correlation coefficient indicates statistically significant dependence of Pb and Cr concentrations in *Mesocestoides* spp. on contents of these heavy metals in the host’s intestine revealing thus a significant positive correlation. On the other hand, correlation between the concentrations of Cd, Cu, Zn, Mn, and Ni in parasites and between the levels of these heavy metals in the host intestine is not significant.

**Figure 3:**
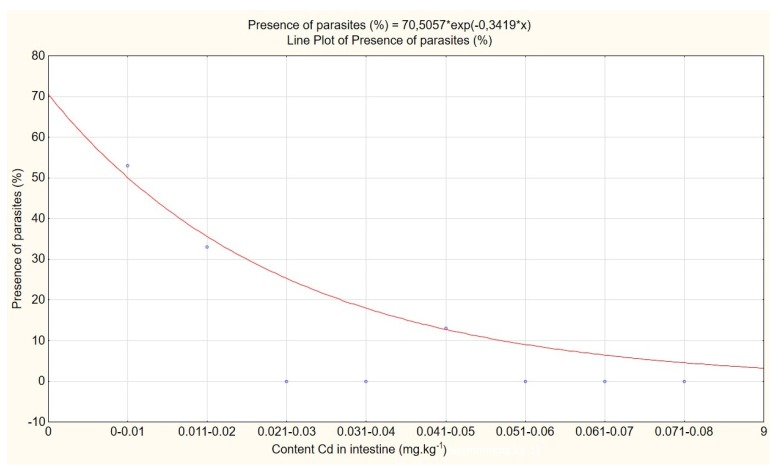
Prevalence of parasites and content of Cd in intestines of the host.

Therefore, occurrence of parasites was related to concentration of Cd in intestines of the host. Prevalence of parasites decreased exponentially with increasing Cd concentrations in the host’s intestine. No parasites were found in the intestines of the hosts that had accumulated Cd in concentrations higher than 0.05 mg kg^−1^ (Figure 3).

## 4. Discussion

High metal accumulation capacity has previously been reported for fish parasites, especially for spiny-headed worms from the phylum Acanthocephala. Concentration of metals in the parasites was up to 2700x higher than that in the tissues of their hosts [16]. The tapeworms of the genus *Hymenolepis*, which parasitize rodents, were also subjected to tests. After exposure to Pb under laboratory conditions, parasites contained the metal in amounts 17 times higher than those found in rat kidneys [7,17].

Similarly, parasites of the genus *Moniliformis* (Acanthocephala) parasitizing in rat intestine contained Cd in concentration 119x higher than the intestines themselves [18]. Sures et al. [19] also investigated the ability of heavy metals to accumulate in *Macracanthorhynchus hirudinaceus* in pigs and found that Cd, Cu, Fe, Mg, Mn, Ni, and Pb concentrations in parasites were up to 160 times higher than the amounts of the above metals in host tissues.

Our measurements confirmed the findings of other authors showing that the parasite tissues contain higher levels of Pb, Cr, Zn, Cu, Mn, and Ni than the tissues of their hosts [16,20,21]. The bio-accumulation factor (BAF) reflecting the ratio of heavy metal level between parasite and host tissues was always greater than 1. The highest accumulation capacity of Pb was found in *Mesocestoides* spp., which accumulated 21 times more Pb than the fox’s small intestine. Moreover, parasites can also accumulate other metals like Cr (2–4.5x) and Mn (2–9x) well. On the contrary, it was found that Cd content in parasite tissues does not grow proportionally to the Cd content in fox tissues such as in small intestine. Some authors report the ability of selected parasites to accumulate significant amounts of elements and to influence their concentrations in the host’s body [1,11,12]. However, based on our results, the above findings cannot be taken for granted. The influence of parasites on the content of Pb, Cr, Cu, Zn, Mn, and Ni in fox intestines was statistically inconclusive, perhaps because of the limited sample size.

Based on the results of our research, we can agree with findings reported in the following studies: parasites neither cause reduction of some heavy metal amounts in the host tissue [22] nor they can serve as natural bioremediators of heavy metals as expressed in [13]. However, content of Cd in a host may show influence on occurrence of parasites in its body.

In accordance with other works, it was verified that red foxes with parasites had lower Cd concentrations in their intestines than red foxes without parasites. However, low Spearman’s rank correlation coefficient shows that Cd concentrations in parasites do not increase proportionally to the Cd content in intestines of the host.

As the concentration of Cd in host intestines increases, prevalence of parasites decreases exponentially. The results of this study show, that parasites may demonstrate higher sensitivity to the toxic effects of Cd than their hosts do. Along with their limited ability to accumulate Cd, higher sensitivity of parasites to its toxic effects can reduce probability of parasitisation, when a certain level of Cd contamination in the host is reached.

The hosts, whose concentration of Cd in intestines exceeded 0.05 mg kg^−1^, did not contain parasites in their intestines. To compare, Regulation (EU) No 488/2014 allows cadmium content in various foods to reach 0.05–0.2 mg kg^−1^ at maximum [23].

According to Sures [1], Cestodes and Acanthocephala parasites meet most of the criteria for ideal toxicity sentinels because they are large, widespread, easy to collect, and they can provide information about the average host exposure in its home district. Undoubtedly, the fox is a suitable host, because in contrast to the rodents they prey upon, it lives in a relatively large territory [24]. Similar models of pollution markers have already been studied [18]. Sures [10] suggests the use of parasites accumulating metals as a pre-concentration step before detection of Pt and Rh in exhaust gases. In Antarctica, Pb and Ag occurrence were studied [25]. Fish intestinal parasites have also been used to investigate heavy metal water pollution in Vaal Dam and Lake Victoria, namely to examine Pb and Ag levels. In both cases, the metals were barely detectable in the hosts, but in their parasites, they could have been easily detected [26,27]. According to Sures [8,16], it is extremely important to employ effective indicators also for urban areas. The above researcher proposes a host-parasite system of rat-*H. diminuta* for urban ecosystems as a usable and promising mean at least for Pb detection because both rats and their parasites are globally widespread and readily available.

Thus, based on the results of this study, the red fox-cestode system in forest ecosystems is suggested to become a possible alternative or complementation to the above-mentioned ones, serving thus as a means of biomonitoring of heavy metals, especially of Pb, Cr, and Mn. Parasites are supposed to be able to detect occurrence of these metals in the environment rather soon, before they accumulate in food sources at a concentration exceeding the hygienic limits.

In our case, samples taken from the animals that were to be killed in hunts were analysed. However, should an extensive large scale research be required, the number of animals killed through wildlife management measures would not be sufficient. Killing foxes only for the purpose of monitoring contamination by heavy metals would certainly raise ethical questions. It is, therefore, appropriate to pay attention to other, especially non-invasive monitoring methods that do not request killing. Animal hair analysis is commonly used for the purpose of environmental pollution monitoring [28,29,30].

A substantial decrease in prevalence of intestinal fox parasites that is associated with increase in Cd concentrations in the host intestine was found in this study. The above finding confirms conclusions of some authors on influence of Cd on occurrence and life functions of other organisms, even at very low concentrations [20,31,32].

Cadmium affects cell proliferation, differentiation, and apoptosis. These processes influence DNA repair mechanism, generation of “reaction oxygen species” and also induction of apoptosis. Cadmium binds to mitochondria. Moreover, it can inhibit both cellular respiration and oxidative phosphorylation even at low concentrations [33]. Thus, Cd threatens biodiversity of different systems in nature, as reported in [34,35,36,37,38,39]. Moreover, it also poses a health risk to humans.

Chronic Cd exposures have been reported to be associated with chronic kidney disease, osteoporosis, diabetes, cardiovascular disease, and with cancer. For common population, food represents the primary source of exposure by Cd. The major food sources that substantially enhance Cd exposure are represented by rice and grains, shellfish and seafood, and meat including edible offal, and vegetables. The estimated weekly intake (WI) values for Cd via rice consumption ranged from 20 to 82 mg Cd per kg bw. In Thailand, most (88.0%) of the people living in urban areas mainly consumed rice grown locally in the contaminated areas [40]. Cadmium pollution has not been reported only in Thailand, it has been detected worldwide, from Scandinavia [41] to Antarctica [42], as well as in the Czech Republic [43]. It is thus regarded as a serious global problem with an impact on stability of ecosystems and on safety of food sources. Safe food sources with reduced cadmium content have been sought to decrease exposure by cadmium [44].

## 5. Conclusions

The results of our study confirm the following findings of other authors: the contents of Cd accumulated in the small intestine of foxes without intestinal parasites are higher than its levels in the small intestine of foxes with intestinal parasites, and the difference in the above Cd amounts is statistically significant.

Statistically significant positive correlation between concentrations of Pb, Cr, and Mn in the intestinal parasites (*Mesocestoides* spp.) of foxes and between levels of these metals in the small intestine of foxes was proved. The correlation between Cd levels in fox intestinal parasites and between Cd amounts in their intestines is statistically inconclusive.

An exponential decrease in prevalence of intestinal parasites (*Mesocestoides* spp.) of foxes that depends on increasing concentration of Cd accumulated in the intestines of foxes was found.

Further research is suggested to elucidate causes of the above findings and to clarify possible impacts of such specific factors on biodiversity or on human health.

## Figures and Tables

**Table 1:** Heavy metal contents in fox intestines and in their parasites.

Tissues		Metal (mg kg^−1^)						
		Cd	Pb	Cr	Cu	Zn	Mn	Ni
Small intestines free of parasites n = 19	x̅	0.03	0.52	0.14	1.37	21.9	4.27	0.23
	x̅_0,5_	0.03	0.29	0.06	1.31	20.7	1.82	0.20
	x̅_R_	0.03	0.34	0.07	1.33	17.8	1.65	0.20
	L_L_	0.02	0.18	0.05	1.05	17.3	1.53	0.13
	L_U_	0.04	0.61	0.24	1.68	23.2	4.09	0.29
Small intestines with parasites n = 15	x̅	0.01	0.22	0.13	1.19	23.9	2.84	0.19
	x̅_0,5_	0.01	0.13	0.09	1.07	24.2	1.77	0.12
	x̅_R_	0.01	0.12	0.09	1.09	23.4	1.89	0.15
	L_L_	0.00	0.06	0.06	0.97	20.9	1.47	0.11
	L_U_	0.01	0.37	0.15	1.24	27.4	2.39	0.26
Nematoda (roundworms) n = 4	x̅	0.08	1.37	0.34	1.65	33.2	4.32	0.30
	x̅_0,5_	0.05	1.23	0.18	1.78	34.1	4.06	0.26
	x̅_R_	0.04	1.23	0.18	1.81	26.9	4.04	0.26
	L_L_	0	0.65	0.12	0.04	13.9	3.09	0.10
	L_U_	0.20	2.25	0.22	2.99	50.6	6.09	0.59
Cestoda (tapeworms) *Mesocestoides* n = 9	x̅	0.09	3.23	0.54	3.77	60.8	26.7	1.13
	x̅_0,5_	0.03	2.34	0.35	2.72	59.0	20.6	0.70
	x̅_R_	0.06	2.46	0.36	2.52	50.3	21.3	0.62
	L_L_	0	0.69	0.07	0	26.2	5.62	0
	L_U_	0.25	4.82	0.98	8.38	97.1	46.0	3.31
Cestoda (tapeworms) n = 4	x̅	0.07	0.83	0.57	8.10	63.2	21.1	4.15
	x̅_0,5_	0.02	0.66	0.42	3.93	55.6	18.0	3.06
	x̅_R_	0.02	0.65	0.40	3.44	42.7	16.8	2.83
	L_L_	0	0.19	0.10	0	11.7	3.15	0
	L_U_	0.28	1.82	1.33	4.59	129.8	39.7	11.26

x̅ arithmetic mean; x̅_0,5_ median; x̅_R_ expressed point estimate of Box-Cox data transformation; L_U_ upper limit of confidence interval of the measure of location for significance level = 0.05. L_L_ lower limit of confidence interval of the measure of location for significance level = 0.05.

**Table 2:** Dependence of heavy metal concentrations in *Mesocestoides* spp. on heavy metal contents in the intestine.

*Mesocestoides* spp. vs. Intestine	Metal						
	Cd	Pb	Cr	Cu	Zn	Mn	Ni
Spearmans’ rank correlation coefficient (r)	0.23	0.75	0.77	0.03	0.32	0.47	0.03
*p*-value	>0.05	<0.05	<0.05	>0.05	>0.05	>0.05	>0.05
